# Analytical Capability of Defocused µ-SORS in the Chemical Interrogation of Thin Turbid Painted Layers

**DOI:** 10.1177/0003702815615345

**Published:** 2016-01

**Authors:** Claudia Conti, Marco Realini, Alessandra Botteon, Chiara Colombo, Sarah Noll, Stephen R. Elliott, Pavel Matousek

**Affiliations:** 1 Institute for the Conservation and Valorization of Cultural Heritage (ICVBC), National Research Council, Milan, Italy; 2 Department of Chemistry, University of Cambridge, Cambridge, UK; 3 Central Laser Facility, Research Complex at Harwell, STFC Rutherford Appleton Laboratory, Harwell, Oxford, UK

**Keywords:** Spatially offset Raman spectroscopy, SORS, Subsurface, Non-destructive, Paintings, Turbid, Diffusel scattering

## Abstract

A recently developed micrometer-scale spatially offset Raman spectroscopy (μ-SORS) method provides a new analytical capability for investigating non-destructively the chemical composition of sub-surface, micrometer-scale thickness, diffusely scattering layers at depths beyond the reach of conventional confocal Raman microscopy. Here, we demonstrate experimentally, for the first time, the capability of μ-SORS to determine whether two detected chemical components originate from two separate layers or whether the two components are mixed together in a single layer. Such information is important in a number of areas, including conservation of cultural heritage objects, and is not available, for highly turbid media, from conventional Raman microscopy, where axial (confocal) scanning is not possible due to an inability to facilitate direct imaging within the highly scattering sample. This application constitutes an additional capability for μ-SORS in addition to its basic capacity to determine the overall chemical make-up of layers in a turbid system.

## Introduction

The recently developed technique of defocused micrometer-scale spatially offset Raman spectroscopy (μ-SORS) provides a new analytical tool for interrogating the chemical make-up of thin stratified layers in highly turbid media.^1^ Such layers are, for example, found in cultural heritage objects, such as painted statues, mural and panel paintings, and other decorated materials,^2^ in areas such as biology, polymer sciences, or the paper industry.^3^ In art, the presence of several stratified layers of paint can originate from the original artist’s work or from multiple restoration processes often applied over many centuries. It is critically important to know the composition of these layers in order to understand the artist’s technique and to be able to apply effective conservation treatments. Due to the uniqueness and high value of art objects, it is often impossible to sample by invasive means (e.g., using cross-sectional analysis with conventional Raman microscopy). In this context, μ-SORS analysis can provide an important new analytical capability, being ultimately potentially fully non-invasive and non-destructive if developed into a portable tool (the current instruments require samples to be brought to a Raman instrument and placed under its microscope objective). Here, we demonstrate experimentally for the first time an additional capability of μ-SORS to determine whether detected chemical compounds either originate from distinct, separate (sub-) layers or whether they are mixed in a single (sub-) layer. Although our previous work demonstrated the capability to determine the presence of layers and the order of stratification, the ability to distinguish between mixed and separate-layer cases has not previously been experimentally verified as it had not been shown that two components mixed in a single layer will not show any relative intensity variation during a µ-SORS defocusing scan. In addition, it has not been established that the relative intensity variation of Raman signals from the two chemical constituents with separate layers or the absence of this variation with mixed layers can be detected even through a third turbid overlayer.

**Figure 1.**
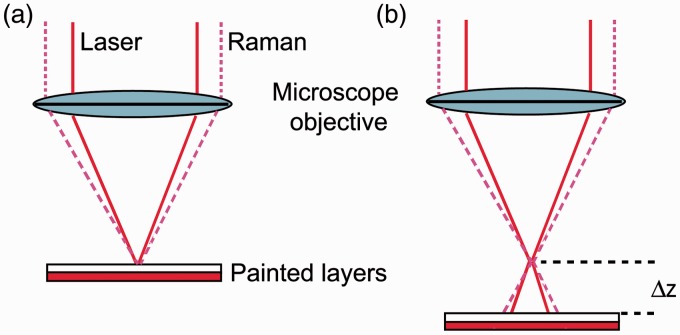
Schematic of the defocused μ-SORS measurement, consisting of acquisitions at: (a) “imaged” and (b) “defocused” positions.

The principle of defocusing μ-SORS has been described in detail earlier.^1–3^ In brief, the concept relies on collecting at least two Raman spectra using a Raman microscope; first, with the sample being in a conventional “imaged” position and then, by moving the sample away from the microscope objective by a “defocusing distance Δz”, in a “defocused” position. The sample displacement leads to the defocusing of both the laser-illumination and Raman-collection zones on the sample surface and their consequent enlargement (Figure 1). The first measurement (the “imaged” position) yields typically a Raman spectrum dominated by the surface layer and corresponds conceptually to a zero-spatially offset measurement in a conventional SORS analysis. The second measurement (“defocused” position) produces a Raman spectrum which has a significantly higher degree of Raman-signal contributions from sub-layers.

The spatial offset on which the SORS process relies^4,5^ is present at a single photon level. Each detected Raman photon can be traced back to its originating laser photon. With extended illumination and collection, some originating laser photons can be spatially separated from the point of emergence from the sample of the corresponding Raman photon, depending on the degree of the overall laser–Raman photon pathway within the medium.^6^ This is in contrast to the “imaged” position measurement where no such separation can be present to any significant degree.

In a two-layer situation, a simple numerical processing involving a scaled subtraction of the “imaged” spectrum from the “defocused” spectrum, and cancelling the contribution of the top layer, can be used to recover the pure Raman spectrum of the sub-layer. The pure Raman spectrum of the top layer can be obtained analogously in a reverse process.

The stratification of the layers can be determined by examining the rate of decay of the Raman intensities of individual chemical components with the degree of defocusing. If two chemically distinct layers are present, their decay rate will be different and the intensity ratio of their corresponding Raman components will vary as a function of the defocusing distance, Δz. On the other hand, if the two chemical components are mixed in a single layer, the decay rates of the Raman bands belonging to the two individual components with defocusing will be identical and no relative change of the Raman band intensities with respect to each other will be present as a function of defocusing. From this it is possible, therefore, to deduce if two pigments are mixed and deposited in a single layer in the sample or if they are present as two separate layers. This capability is demonstrated here experimentally for two situations: (1) the pigments are deposited as the outermost surface layer(s) (individually or mixed), and (2) when the layer(s) containing the pigments (deposited individually or mixed) is obscured by another turbid overlayer.

## Experimental

**Figure 2.**
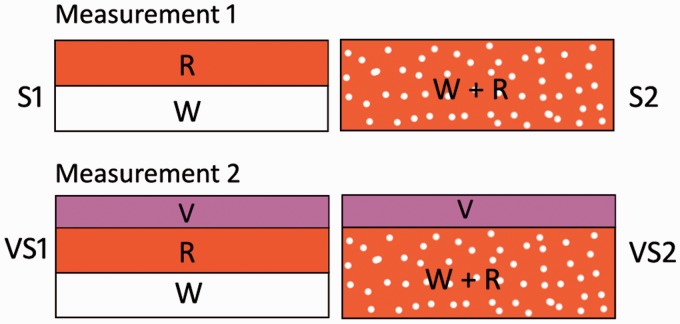
Schematic diagrams of turbid stratified samples used in the study.

The specimens consist of painted layers simulating a real artistic stratigraphy (Figure 2). Two common pigments were used, red ochre (hematite – Fe_2_O_3_) and titanium white (rutile – TiO_2_) here called “R” (red) and “W” (white), respectively. The specimen S1 consists of an “R” layer (50 µm thick) on a “W” layer (50 µm thick); the specimen S2 was prepared by mixing rutile and hematite in a 1:10 ratio; the thickness of the layer was 100 µm. The specimens were prepared in an attempt to obtain semi-homogeneous layers, both in terms of thickness and distribution of the pigment within the layer. Both S1 and S2 were spread on a substrate consisting of a yellow layer (consisting of bismuth vanadate, – BiVO_4_) deposited on a paper sheet. The yellow layer allows one to avoid the overlapping of W with the white rutile pigment component of the paper surface.

Further sets of measurements were carried out by placing a turbid 40 µm thick layer of cobalt violet pigment (V, violet) on top of S1 and S2 (VS1 and VS2 specimens, respectively).

The μ-SORS measurements were carried out using a Senterra dispersive μ-Raman spectrometer (Bruker) with a 1200 grooves/mm grating and 20 × objective of an Olympus BX51 microscope. The laser excitation wavelength was 785 nm with a power at the sample of up to ∼100 mW. The Raman spectra were acquired using a Peltier-cooled CCD detector (1024 × 256 pixels). No confocal pinhole was used in any of the measurements. The spectra were acquired with a 150 s acquisition time (five accumulations, 30 s each).

Cross-sections of S1 and S2 specimens were prepared, observed in reflected light using a Leitz Ortholux microscope with an Ultropack illuminator equipped with a digital image-capturing system, and mapped by Raman spectroscopy to confirm composition and homogeneity of the layers. A Senterra dispersive μ-Raman spectrometer (Bruker) was employed also to acquire maps using a 1200 grooves/mm grating and 785 nm laser-excitation wavelength. The power at the sample was 25 mW and spectra were collected with a 50 × objective, with a step size between 10 and 15 µm along the *x*- and *y*-axes, an exposure time of 1 s, and with four accumulations.

The Raman intensities of individual layers were derived using multivariate curve resolution (MCR) analysis from the spectral region of 340–540 cm^−1^ containing the Raman bands of all three potential layers (V, R, and W). The analysis was performed using the Eigenvector Solo 7.9.5 software suite (Eigenvector Research Inc., Manson, WA) and the pre-processing steps consisted of baseline removal (Whittaker filter) followed by spectral normalization to the area of the entire spectrum within the analyzed range. Pure spectral components of individual layers were added to the dataset to guide the analysis that was carried out with non-negativity constraints. The number of components used in the MCR analysis was set to 3.

## Results and Discussion

Two types of measurements were performed here (Figure 2). First, a two-chemical component system was interrogated when deposited as two distinct layers or when mixed homogeneously in a single layer (Measurement I). Second, the same measurements were repeated when the layers were both located beneath another turbid layer (cobalt violet pigment) (Measurement II).

### Measurement I

**Figure 3.**
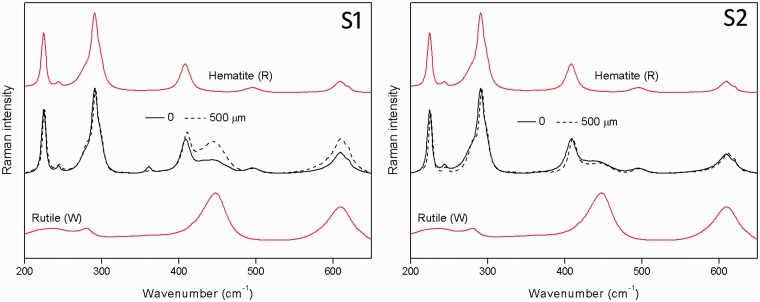
Baseline-corrected Raman spectra normalized to the intensity of the 224 cm^−1^ band of hematite for extreme defocusing positions (0 and 500 µm) for two-layer (S1) and mixed single-layer (S2) systems. The pure Raman spectra of individual pigments are shown for comparison. The spectra are offset for clarity.

The μ-SORS spectra from the measurements performed on S1 and S2 systems are shown in Figure 3 for two extreme defocusing positions (“imaged” and 500 µm “defocused” sample displacement). The spectra are normalized to the maximum Raman band intensity to visualize the relative intensity changes between the two pigments used in the study. The mixed layer (S2) spectra show very little change between the relative intensities whereas the two-layer system (S1) exhibits a significant change in relative intensity between the two defocusing positions, in line with expectations. In particular, the two characteristic Raman bands of rutile at 446 and 611 cm^−1^ strongly increase at 500 µm defocusing. The order of the layer is identifiable from the measurement, with the red pigment diminished in intensity more with the introduction of defocusing, indicating that this layer is located above the white layer.

**Figure 4.**
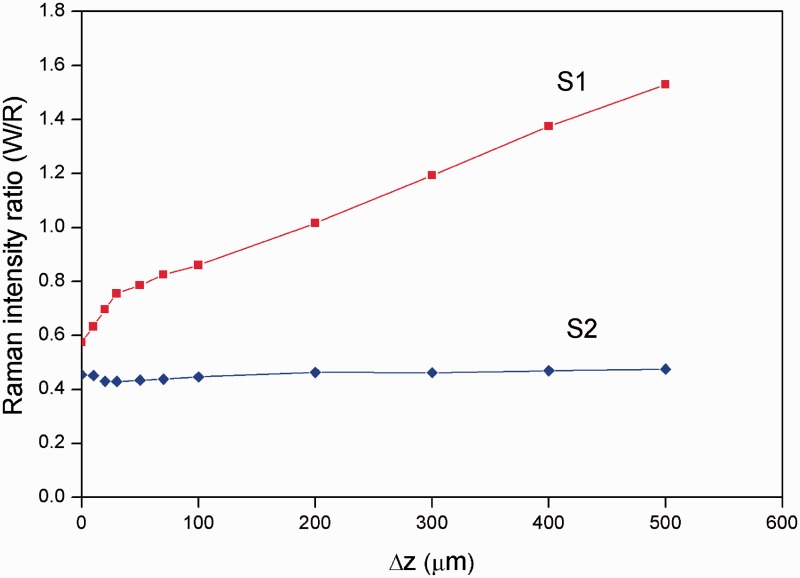
Raman-intensity ratio between the MCR components corresponding to W and R constituents for two-layer (S1) and mixed single-layer (S2) systems on the extent of defocus.

The Raman intensity dependence of individual signals is shown in full in Figure 4.

**Figure 5.**
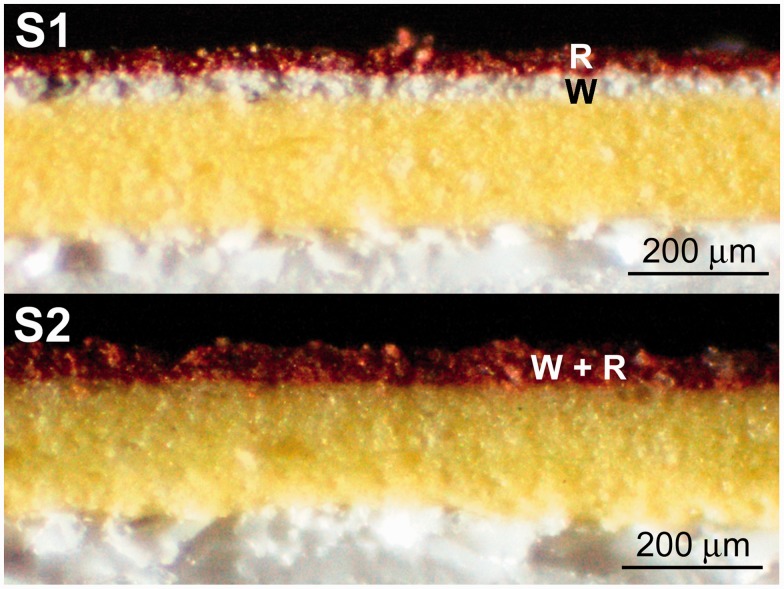
Optical-microscopy images of the specimen cross-sections. In S1, the pigments are spread in two different layers with R on the top; in S2, the two pigments are mixed in a single layer. A yellow layer and white paper were used as substrates for both the specimens.

**Figure 6.**
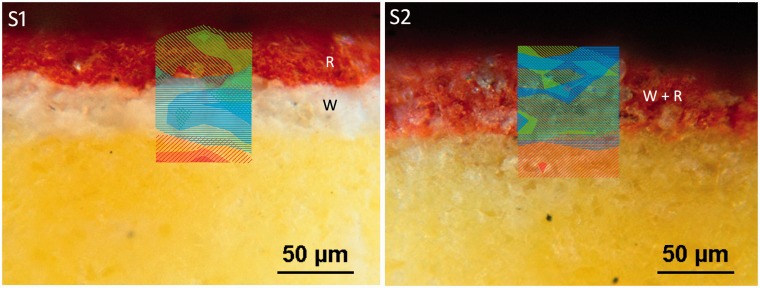
Raman maps of the distribution of rutile (blue color) and hematite (green color) superimposed on micrograph images of S1 and S2 sample cross-sections. For the sake of completeness, the yellow substrate was also mapped (red color).

Cross-sectional analysis confirmed the high homogeneity in terms of composition and thickness of the specimens (Figures 5 and 6). The strongest bands were mapped, namely 446 cm^−1^ and 292 cm^−1^ for rutile and hematite, respectively (see Figure 6).

### Measurement II

**Figure 7.**
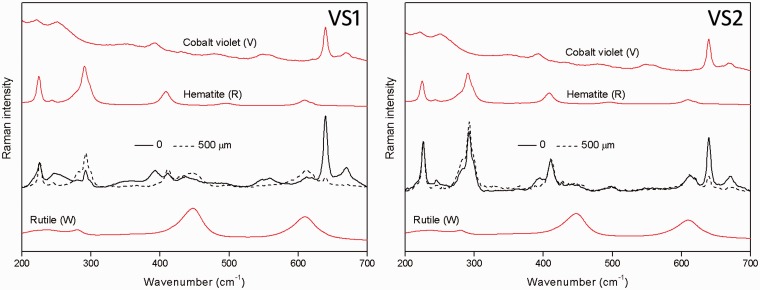
Baseline-corrected Raman spectra normalized to the intensity of the 224 cm^−1^ band of hematite for extreme defocusing positions (0 and 500 µm) for the two-layer system (VS1) and mixed single-layer system (VS2), both located under a 40 µm thick layer of cobalt violet. The pure Raman spectra of individual pigments and cobalt violet are shown for comparison. The spectra are offset for clarity.

**Figure 8.**
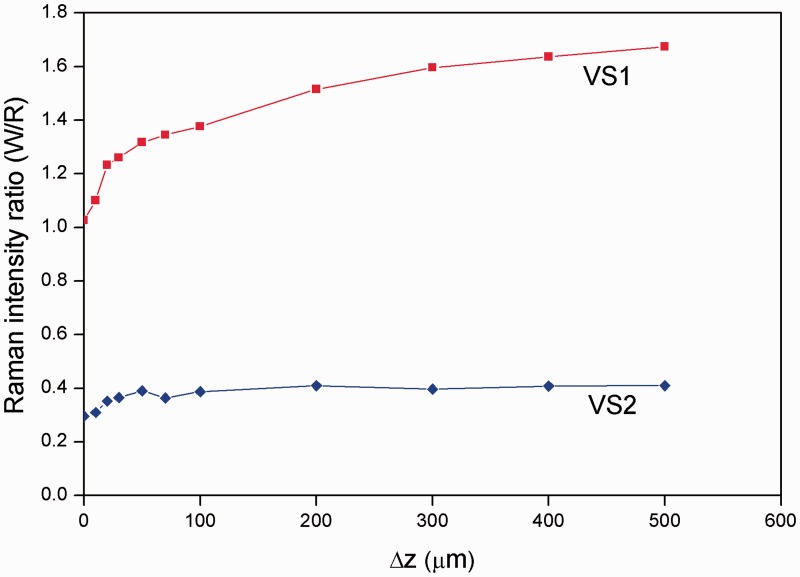
Raman-intensity ratio between W and R of the two-layer (VS1) and mixed single-layer (VS2) systems as a function of defocus when obscured by a third turbid overlayer (“V”).

The measurement was repeated on the identical systems when located under a 40 µm thick layer of cobalt violet pigment. The overlapped spectra again exhibit an approximately constant intensity ratio between the two pigments with defocusing distance Δz for the mixed-layer system (VS2), but this intensity ratio varies for the pigments deposited as two distinctly different layers (VS1) (Figure 7). The order of the layers is again identifiable from the measurement, even if they are located under a turbid layer consisting of cobalt violet, with the red component decaying faster with the displacement distance Δz, indicating that this layer is located above the white layer. The Raman intensity dependence of individual signals is shown in full in Figure 8.

## Conclusions

The capability of μ-SORS to interrogate stratified layers and determine whether chemical compounds are deposited in distinct layers or mixed in a single layer has been demonstrated here for both the outermost surface layers and when obscured by another turbid overlayer. This is determined by monitoring the evolution of the relative Raman intensities of the components concerned as a function of vertical (z-) sample displacement (defocus). The application of these outcomes to a number of areas, including conservation of cultural heritage objects, provides a novel, non-invasive approach for the selective depth exploration of multilayer, highly turbid thin systems, where conventional Raman microscopy cannot be used to obtain direct imaging of inner sample components. This provides an additional analytical capability of μ-SORS, in addition to its basic ability to determine the overall chemical make-up of layers in stratified turbid systems.
